# Bilateral Undescended Testes With Unilateral Torsion Presenting As Acute Abdomen: A Case Report

**DOI:** 10.7759/cureus.81501

**Published:** 2025-03-31

**Authors:** Muhammad Mudasir Saleem, Mishal Pervaiz, Maham Sultan, Sahab Nasir Tiwana, Ismail Mazhar, Uswah Shoaib, Javaria Ahmad, Zartaj Ghaffar

**Affiliations:** 1 Pediatric and General Surgeon, Combined Military Hospital (CMH), Lahore, PAK; 2 Anesthesia, Punjab Rangers Teaching Hospital, Lahore, PAK; 3 Pediatric Surgery, Combined Military Hospital (CMH) Lahore Medical College and Institute of Dentistry, Lahore, PAK

**Keywords:** orchidectomy, testis, torsion, undescended, unilateral

## Abstract

Undescended testis (UDT) is the most common congenital urological anomaly. The primary concerns warranting surgical intervention include an increased risk of malignancy, infertility, and testicular torsion. Although testicular torsion is rare, it is a serious complication that can lead to ischemic injury and organ loss if not promptly diagnosed and managed. Bilateral undiagnosed UDT with torsion in an ipsilateral palpable testis is even more rarely reported. We present a similar case of a six-year-old male with bilateral undescended testes--the left being intra-abdominal and the right located in the inguinal canal with torsion--presenting with features of an acute abdomen. He was managed in a single procedure with left laparoscopic orchidopexy and right orchiectomy due to a non-salvageable ischemic testis.

## Introduction

Undescended testis (UDT) results from the failure of one or both testes to fully descend into the scrotal sac during embryogenesis. It affects approximately 1.6% to 9.0% of newborn males, with prematurity and low birth weight being the leading etiological factors [1]. UDTs can be located anywhere along the normal route of descent, either in the inguinal canal (palpable) or the abdominal cavity (nonpalpable) [2]. Torsion in UDT is a rare complication, often caused by abnormal gubernacular attachment or forceful cremasteric muscle contraction, unlike the bell-clapper deformity associated with testicular torsion in descended testes [3]. Testicular torsion is a surgical emergency due to the risk of ischemia and necrosis, which increases with delayed diagnosis and intervention. Timely recognition and urgent surgical management are crucial to prevent organ loss, which can have significant psychological and emotional effects on affected children [4].

Here, we report a rare case of bilateral UDT with right-sided torsion and a left-sided abdominal testis, posing both diagnostic and therapeutic challenges. The patient was managed with a right orchiectomy and simultaneous laparoscopic orchidopexy for the left testis to preserve fertility and minimize the psychological impact of losing the contralateral organ.

## Case presentation

**Figure 1:**
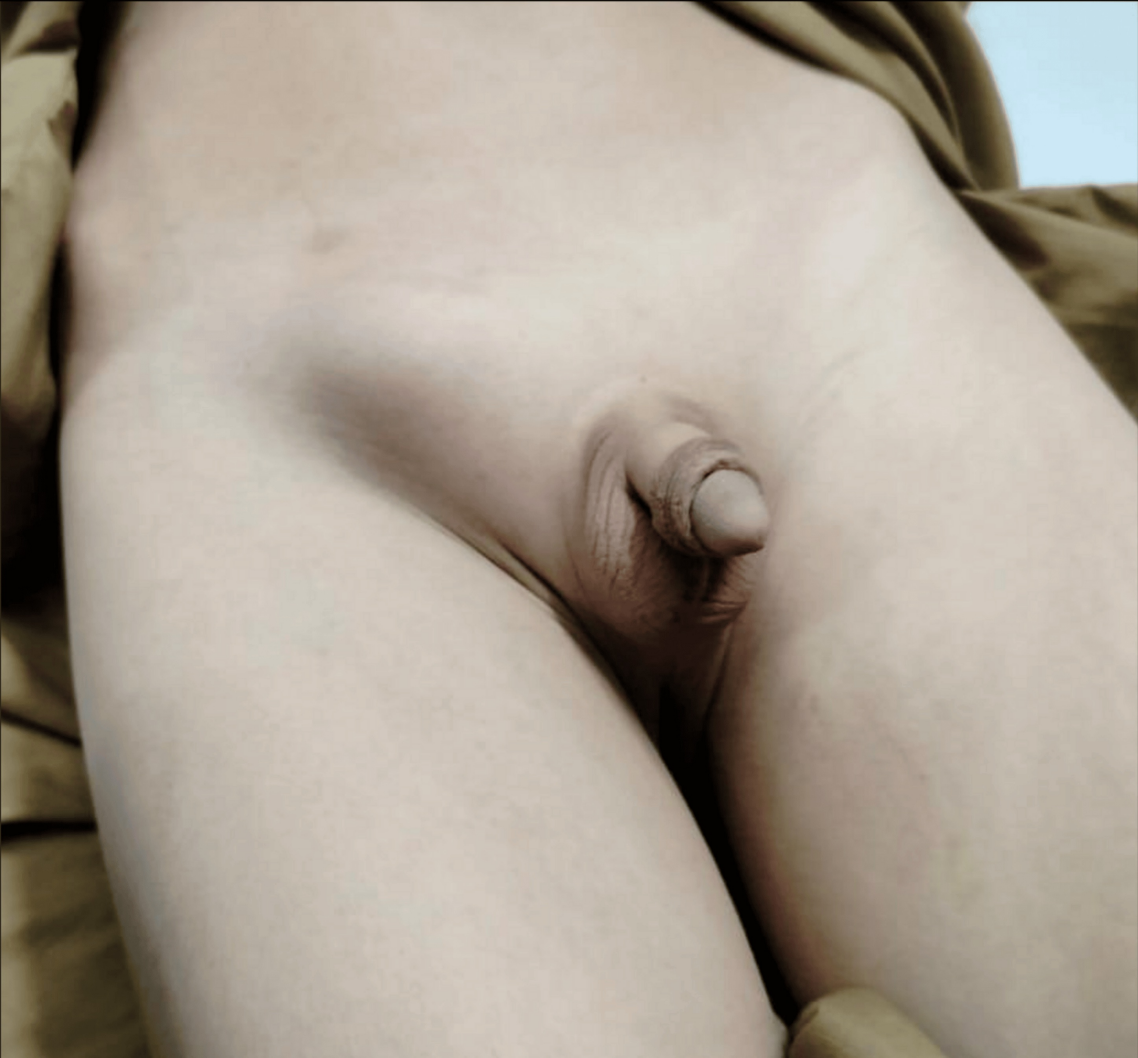
Bilateral empty scrotum with right groin swelling on presentation.

A six-year-old male presented to the emergency department with a three-day history of progressively worsening, excruciating pain in the right lower abdomen, accompanied by nausea and vomiting. There was no history of fever, trauma, or urinary or bowel complaints. The child had previously visited local practitioners and was prescribed oral analgesics, but his symptoms did not improve. The birth history was unremarkable, with no history of prematurity. On general physical examination, his vitals were normal. Abdominal examination revealed a soft, non-distended, and non-tender abdomen across all quadrants. Groin's examination identified a 4×4 cm hard, tender, and firm lump in the right mid-inguinal canal, with an empty right hemiscrotum. Further evaluation revealed an empty left hemiscrotum, with no palpable testis in the left inguinal canal, superficial inguinal pouch, or perineum (Figure 1).

**Figure 2:**
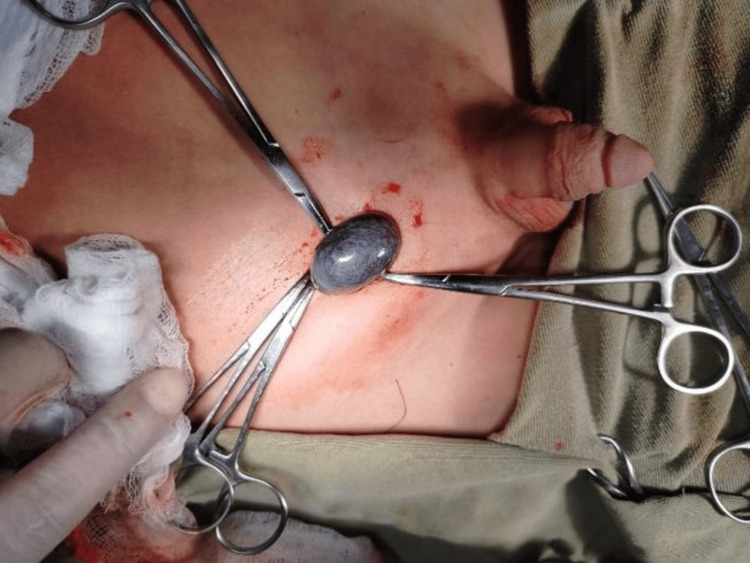
Groin exploration showing necrotic testicular mass.

Given the high index of suspicion, further workup was initiated, including baseline investigations and Doppler ultrasound of the right groin, along with an ultrasound of the abdomen and left groin to locate the left testis. Doppler ultrasound revealed a testicular mass in the right groin with no blood supply, while the ultrasound of the abdomen and left groin failed to identify the left testis. Emergency exploration of the right groin and a diagnostic laparoscopy for the nonpalpable left testis were planned, and the patient was optimized for surgery. Informed written consent was obtained for a right orchiectomy after a detailed explanation. A small skin crease incision was made for right groin exploration, which revealed a necrotic, bluish-discolored testis (Figure 2). 

**Figure 3:**
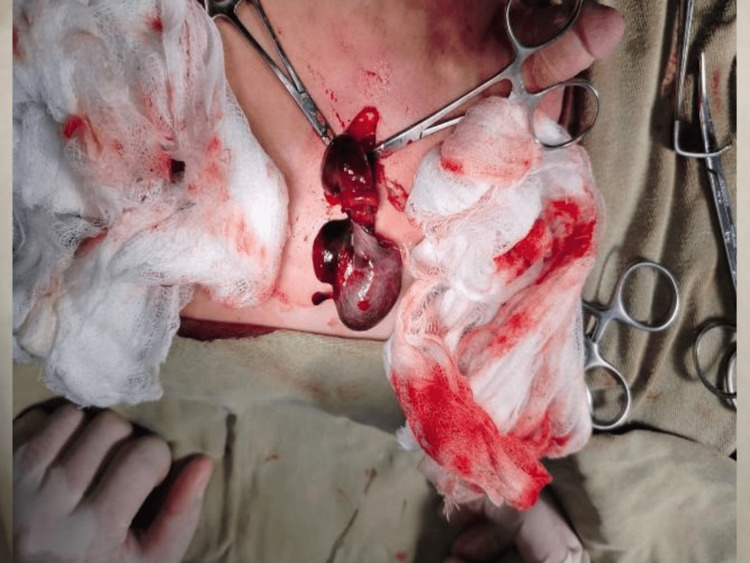
Necrotic testis with twisted, congested spermatic cord.

Further delivery of the cord into the wound revealed a twisted cord (Figure 3). Detorsion did not improve the testicular color, necessitating orchiectomy. The cord was ligated at a high level, and the wound was closed in layers.

**Figure 4:**
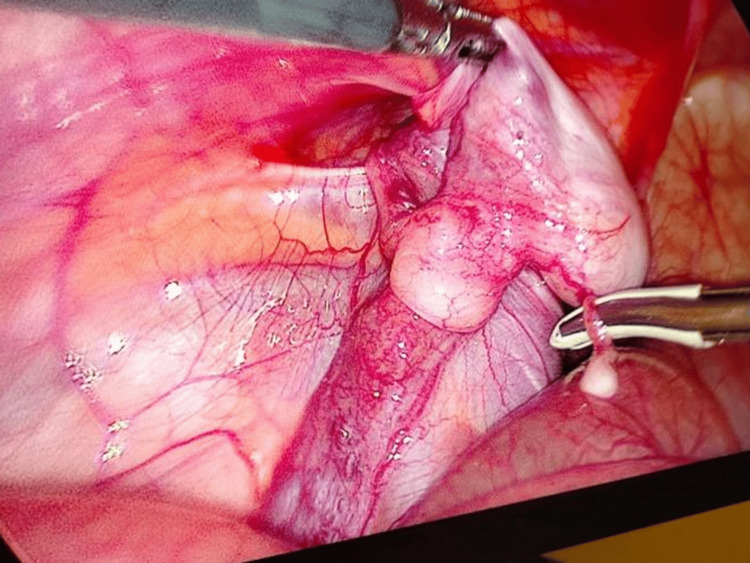
Diagnostic laparoscopy showing left intra-abdominal testis close to the deep ring.

The pneumoperitoneum was established through a 5 mm umbilical port to locate the left testis, maintaining an intra-abdominal pressure of 10-12 mmHg. The left testis was identified near the left deep ring (Figure 4).

**Figure 5:**
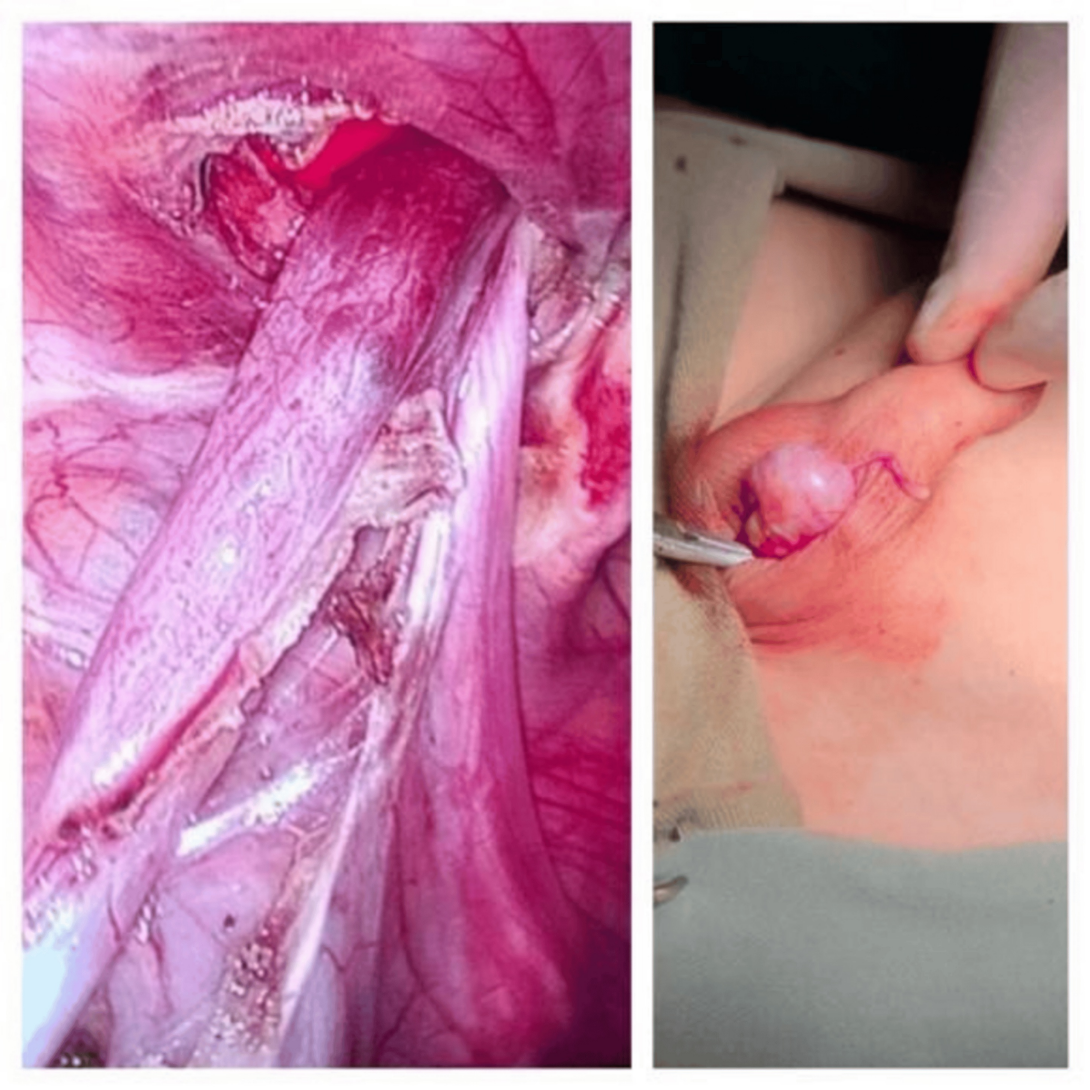
Intraoperative picture showing mobilized spermatic vessels and vas deferens with final placement in subdartos pouch.

Two 5 mm working ports were placed in the right and left upper quadrants. The testis was mobilized by dividing the gubernaculum. Further dissection along the spermatic vessels and vas deferens provided sufficient length, allowing the left testis to reach the right deep ring without tension. A tunnel was created medial to the lateral umbilical ligament, and the testis was brought down into the left hemiscrotum and secured in a subdartos pouch (Figure 5).

The child had an uneventful postoperative recovery and was discharged on the third postoperative day. Follow-up at one and three months showed a well-healed wound, with the left testis palpable in the mid-scrotum. The parents were advised to ensure regular follow-ups into adulthood to monitor testicular volume and detect any potential complications early.

## Discussion

Testicular descent from the abdomen to the scrotum is a complex, multistage process that is usually completed by the 35th week of gestation. The pathophysiology of undescended testis (UDT), whether unilateral or bilateral, palpable or nonpalpable, is not fully understood but is believed to be multifactorial, involving genetic, environmental, and hormonal factors [5]. The first reported case of torsion in UDT was described by Delasiauve in 1840 [6]. While testicular torsion can occur in descended testes in both neonatal and prepubertal ages, UDT has a tenfold higher risk of developing torsion [7]. A literature review revealed that torsion can occur in both palpable and nonpalpable UDT, though it is more commonly reported in the palpable variety [8].

The clinical presentation of UDT torsion is often nonspecific, including groin or abdominal pain, poor oral intake, vomiting, and restlessness. Physical examination may reveal inguinal swelling and erythema with a firm, tender mass in the inguinal region and an empty scrotum. As a surgical emergency, UDT torsion can be readily confirmed with Doppler ultrasound, which shows absent or reduced blood flow, as seen in our case. In nonpalpable UDT, diagnostic laparoscopy can confirm and manage torsion [9].

Our patient presented with progressively worsening, severe right lower abdominal pain. The diagnosis was made based on a thorough abdominal and groin examination and confirmed by Doppler ultrasound, which showed an absence of blood flow to the affected testis. This highlights the critical importance of a groin examination in all children presenting with an acute abdomen, as inadequate assessment can lead to misdiagnosis, such as acute appendicitis or an incarcerated inguinal hernia.

Testicular torsion, whether in descended or undescended testes, is a surgical emergency. Studies indicate a poor salvage rate for UDT torsion (up to 10%) compared to torsion in descended testes (up to 90%), primarily due to delayed diagnosis resulting from vague symptoms [10]. Singal et al. reported a similar case of bilateral UDT torsion in a four-month-old infant, where left orchiectomy was performed for the palpable testis, followed by laparoscopic orchidopexy of the contralateral testis at six months [7]. In our case, we opted for contralateral orchidopexy in the same procedure, given the delayed presentation, to avoid future exposure to general anesthesia. This approach also aimed to spare the child from unnecessary physical and psychological trauma associated with repeated procedures.

Additional case reports further illustrate the challenges in diagnosing and managing torsion in UDT. For instance, a 17-year-old male with cerebral palsy presented with a painful right inguinal mass, later identified intraoperatively as torsion of an undescended testicle, leading to orchiectomy [11]. Another case involved a patient with an undescended left testis presenting with acute abdominal pain; imaging confirmed torsion and necrosis, necessitating surgical intervention [3]. These cases underscore the importance of considering testicular torsion in patients with undescended testes who present with acute inguinal or abdominal pain. Prompt imaging and surgical intervention are vital in preventing complications [12]. In summary, torsion of an undescended testis is a rare but serious condition requiring a high suspicion index for timely diagnosis and management. Early recognition and intervention are crucial to prevent adverse outcomes, including testicular loss and compromised fertility.

## Conclusions

In conclusion, this rare case of bilateral undescended testes (UDT) with ipsilateral torsion highlights its unpredictable nature and potential for acute emergencies. It underscores the importance of clinical vigilance and comprehensive preoperative evaluation, as torsion in UDT may not always present with clear symptoms. Torsion poses a silent threat, jeopardizing fertility and increasing the risk of ischemic damage. Clinicians should maintain a high index of suspicion, as timely intervention is crucial for optimizing long-term reproductive and hormonal outcomes. Additionally, nonpalpable UDT must be promptly diagnosed and managed. This case serves as a strong reminder that early diagnosis and proactive surgical intervention are essential to prevent catastrophic outcomes. It also reinforces that an abdominal examination is incomplete without a thorough groin examination to avoid missing critical clinical findings.
